# A retrospective study of Pupingqinghua prescription versus Lianhuaqingwen in Chinese participants infected with SARS-CoV-2 Omicron variants

**DOI:** 10.3389/fphar.2022.988524

**Published:** 2022-10-07

**Authors:** Yidan Dong, Wei Zhai, Bangjiang Fang, Chenyang Liu, Suyun Yuan, Youhua Wang, Qixiang Song, Hai Li, Bin Chen, Dan Cui, Jun Wang, Qiong Wu, Chang Zhou, Maolin Zhou, Shuchun Li, Xu Zhuang, Qingrong Xu, Yu Zheng, Yingen Wu, Junhua Zheng, Min Cao

**Affiliations:** ^1^ Longhua Hospital, Shanghai University of Traditional Chinese Medicine, Shanghai, China; ^2^ Renji Hospital, School of Medicine in Shanghai Jiao Tong University, Shanghai, China

**Keywords:** pupingqinghua prescription, COVID-19, lianhuaqingwen, traditional Chinese medicine, retrospective study

## Abstract

**Background:** Coronavirus disease (COVID-19) seriously endangers global public health. Pupingqinghua prescription (PPQH) is an herbal formula from traditional Chinese medicine used for treatment of SARS-CoV-2 infection. This study aims to evaluate the clinical efficacy and safety of PPQH in Chinese participants infected with the SARS-CoV-2 Omicron variant.

**Methods:** A total of 873 SARS-CoV-2 (Omicron)-infected patients were included. Among them, the patients were divided into the PPQH group (653 cases) and LHQW group (220 cases) according to different medications. The effectiveness indicators (hematological indicators, Ct values of novel Coronavirus nucleic acid tests, and viral load-shedding time) and safety indicators (liver and kidney function and adverse events) were analyzed.

**Results:** There was no significant difference in baseline characteristics between the PPQH group and the LHQW group, except the gender; After the treatment, the levels of IL-5, IL-6, IL-10, NK cells, and INF-α of the patients in the PPQH group showed a downward trend (*p* < 0.05); The viral load shedding time was 5.0 (5.0, 7.0) in the PPQH group and 5.0 (4.0, 7.0) in the LHQW group; both PPQH and LHQW can shorten the duration of symptoms of fever, cough, and sore throat. The re-positive rate of COVID-19 test was 1.5 % in the PPQH group and 2.3 % in the LHQW group. In terms of safety, the levels of γ-GTT decreased significantly (*p* < 0.01); gastrointestinal reaction was the primary adverse reaction, and the reaction rate was 4.7 % in the PPQH group and 9.5 % in the LHQW group.

**Conclusion:** PPQH can shorten the length of hospital stay and improve clinical symptoms of patients with SARS-COV-2 (Omicron), and it also has a good safety profile.

## 1 Introduction

As of 17 June, 2022, the Coronavirus disease (COVID-19) has spread globally since its detection in 2019. Globally, there have been 535,248,141 confirmed cases of COVID-19 and 6,313,229 deaths (World Health Organization, 2022),^,^ which has seriously endangered global public health and caused significant impact and damage to economic development and social stability of all countries.

Currently, five main variants are noteworthy for the Variant of Concern (VOC) by the WHO, including α (alpha), β (beta), γ (gamma), delta (delta), and Omicron. Among them, Omicron is the most prevalent strain in China. It is the most mutated strain of any SARS-COV-2 variant, some of which are associated with transmissibility, disease severity, and immune escape (He et al., 2021; Huang et al., 2022). However, data from the world’s first Omicron serum neutralization trial showed a 22-fold decrease in neutralization activity against the Omicron mutant in the serum of vaccinators compared with the original strain (Cele et al., 2021). These results suggested that the novel Coronavirus vaccine may not be as effective in protecting against Omicron variants. In addition, specific drugs are still lacking in treatment of COVID-19. Antiviral drugs such as paxlovid and VV116 are commonly used in clinics (Lu, 2020; Ng et al., 2022; Shen et al., 2022). However, efficacy and safety of these drugs need further clinical validation. While COVID-19 control still faces multiple challenges in the short term, there is an urgent need to develop more targeted, specific drugs.

Integrated traditional Chinese and Western medicine is widely used in China (Xu et al., 2022). More than 85 % of COVID-19 patients have used TCM therapy, with an effective rate of over 90 % (Yang et al., 2020), showing enormous advantages of TCM in treatment of COVID-19 (Gajewski et al., 2021; Wu and Zhong, 2021). Lianhuaqingwen (LHQH) is recommended as a Chinese patent medicine in the Guideline for the Diagnosis and Treatment of COVID-19 (On Trials, the Ninth Edition) issued by the National Health Commission of the People’s Republic of China (Xu et al., 2022). The combination of drugs can significantly relieve the main symptoms of COVID-19 and shorten the course of the disease, playing a positive role in the treatment of COVID-19 (Hu et al., 2021).

Based on the pathogenesis of the SARS-CoV-2 infection (Omicron), combined with the characteristics of the hot and damp climate in Shanghai, we proposed Pupingqinghua prescription (PPQH) to treat the disease. The formula is composed of *Taraxacum sect. Taraxacum F.H.Wigg. [*Asteraceae*]* (Pugongying), *Spirodela polyrhiza (L.) Schleid. [*Araceae*]* (Fuping), *Atractylodes lancea (Thunb.) DC. [*Asteraceae*]* (Cangshu),*Rheum palmatum L. [*Polygonaceae*]* (Dahuang), *Gardenia jasminoides J. Ellis [*Rubiaceae*]* (Zhizi), *Swertia chirayita (Roxb.) H. Karst. [*Gentianaceae*]* (Yinchen), *Lonicera japonica Thunb. [*Caprifoliaceae*]* (Rendongteng), *Curcuma longa L. [*Zingiberaceae*]* (Yujin), and *Astragalus mongholicus Bunge [*Fabaceae*]* (Huangqi). It has the effect of clearing heat and detoxification, dehumidification, and turbidity. The purpose of this research was to evaluate the clinical efficacy and safety of PPQH in Chinese participants infected with the SARS-CoV-2 Omicron variant.

## 2 Methods

### 2.1 Research object and case source

This retrospective study is carried out to evaluate the clinical efficacy and safety of PPQH in Chinese participants infected with the SARS-CoV-2 Omicron variant.

Patients were confirmed to have SARS-CoV-2 infection by real-time PCR from 20 March, 2022, to 10 June, 2022, in the mobile cabin hospital of Shanghai New International Expo Center.

### 2.2 Diagnostic criteria

The diagnostic criteria follow the Guideline for the Diagnosis and Treatment of COVID-19 (On Trials, the Ninth Edition) issued by the National Health Commission of the People’s Republic of China (Xu et al., 2022).

### 2.3 Included in the standard

(1) Novel Coronavirus infection confirmed by real-time PCR (Ct value > 35 for both ORF1ab and N gene);
(2) Diagnosed with mild COVID-19 according to the diagnostic criteria.
(3) Aged 18–75, regardless of gender.

### 2.4 Exclusion criteria

(1) Patients who were diagnosed with moderate, severe, or critical COVID-19;
(2) Pregnant or lactating women with ECOG scores greater than or equal to Grade 2;
(3) Patients with abnormal liver and kidney function.
(4) Patients who underwent major surgery within 28 days before admission.
(5) Patients with uncontrolled and active bleeding or bleeding tendencies.
(6) Clinically significant gastrointestinal abnormalities that may affect study drug absorption or increase the risk of bleeding or perforation and any previous history of gastrointestinal perforation.
(7) Other malignant tumors that have been requiring treatment in the past 3 years.
(8) Poorly controlled hypertension, defined as systolic blood pressure ≥160 or diastolic blood pressure ≥100 mmHg. Anti-hypertensive drugs and rescreening are permitted.
(9) New York Heart Association Class III or IV congestive heart failure.
(10) Uncontrolled co-morbidities will affect subjects’ compliance with the study requirements.

### 2.5 Medication

1) PPQH group: PPQH is composed of *Taraxacum sect. Taraxacum F.H.Wigg. [*Asteraceae*]* (Pugongying) 30 g, *Spirodela polyrhiza (L.) Schleid. [*Araceae*]* (Fuping) 12 g, *Atractylodes lancea (Thunb.) DC. [*Asteraceae*]* (Cangshu)12 g, *Rheum palmatum L. [*Polygonaceae*]* (Dahuang) 6 g, *Gardenia jasminoides J. Ellis [*Rubiaceae*]* (Zhizi) 12 g, *Swertia chirayita (Roxb.) H. Karst. [*Gentianaceae*]* (Yinchen) 30 g, *Lonicera japonica Thunb. [*Caprifoliaceae*]* (Rendongteng) 15 g, *Curcuma longa L. [*Zingiberaceae*]* (Yujin) 12 g, and *Astragalus mongholicus Bunge [*Fabaceae*]* (Huangqi) 30 g. The drug is prepared by the pharmacy department of the cabin hospital, decocted to 200 ml. Administer 200 ml a day immediately after diagnosis and to be taken twice in the morning and evening.

2) LHQW group: LHQW granules (Shijiazhuang Yiling Pharmaceutical Co., LTD. National drug approval word Z20040063, 6 g/bag). Administer 36g/200 ml a day immediately after diagnosis and to be taken thrice in the morning, noon, and evening.

### 2.6 Observation

#### 2.6.1 Demographic and general indicators

Gender, age, BMI, vaccination history, smoking history, etc.

#### 2.6.2 Validity indicators

(1) Hematological indicators and inflammatory factors: IL-1β, IL-2, IL-4, IL-5, IL-6, IL-8, IL-10, IL-12P70, and IL-17A; lymphocyte subsets: NK cells, Th cells, Ts cells, B cells, TNF-α, INF-α, and INF-γ;
(2) Ct values of the ORF1ab and N genes;
(3) Viral load shedding time: the time from admission after treatment to the last three consecutive negative COVID-19 test results. The negative result of the COVID-19 test is defined as Ct value > 35.

#### 2.6.3 Safety indicators

(1) Laboratory indicators: physical and chemical indicators such as liver and kidney function before and after treatment;
(2) Adverse events: the incidence of adverse events in patients.

### 2.7 Statistical analysis

SPSS 24.0 was used for statistical analysis. Count (percentage) was adopted for summarizing the categorical variables and compared with Chi-square tests. Continuous variables were presented with mean ± standard deviation and compared with independent *t*-test or Wilcoxon rank-sum test. The time to events was presented as the median duration and 95 % confidence interval (95 % CI) and analyzed with Kaplan–Meier analysis. All statistical testing was two-sided, with *p* < 0.05 considered statistically significant.

## 3 Results

### 3.1 Baseline characteristics

A total of 873 patients were included, and among them, 653 cases were in the PPQH group and 220 cases in the LHQW group. Of all patients included, 569 (65.2 %) were female, and 304 (34.8 %) were male; there was a statistical difference in gender between the two groups at the baseline, which may be related to the included cases. The median age was 45.0. The median BMI was 23.8; 790 (90.5 %) patients were vaccinated, of whom, 502 (57.5 %) patients completed the booster vaccination. In terms of symptoms, 152 (17.4 %) patients had fever, 322 (36.9 %) patients had cough, 157 (18.0 %) patients had sore throat, 46 (5.3 %) patients had nasal obstruction or running, 83 (9.5 %) patients had headache or myalgia, and 19 (2.2 %) patients showed fatigue symptoms. In terms of basic diseases, a total of 159 (18.2 %) patients were suffering from basic diseases, among which, 77 (8.8 %) cases were hypertensive patients, 30 (3.4 %) cases were diabetes patients, and 44 (5.0 %) cases were chronic bronchitis patients. There were 241 (27.6 %) patients with smoking history. The population data, vaccination history, clinical symptoms, basic disease history, and smoking history of the PPQH group and LHQW group were consistent (*p* > 0.05). (table 1).

**TABLE 1:** Comparison of the baseline clinical data between the two groups of SARS-CoV-2-infected patients.

	Total (n = 873)	PPQH group (n = 653)	LHQW group (n = 220)	*p* value
Gender, n (%)				< 0.05
Male respondent	304 (34.8)	116 (17.8)	188 (85.5)	
Female respondent	569 (65.2)	537 (82.2)	32 (14.5)	
Age, median (IQR)	45.0 (33.0, 54.0)	45.0 (33.0, 54.0)	43.0 (33.0, 55.2)	0.911
BMI, median (IQR)	23.8 (21.4, 25.9)	23.7 (21.3, 25.9)	24.0 (21.7, 25.9)	0.42
Vaccinated or not, n (%)				0.492
No	83 (9.5)	59 (9)	24 (10.9)	
Yes	790 (90.5)	594 (91)	196 (89.1)	
Booster vaccine administered or not, n (%)				0.874
No	371 (42.5)	276 (42.3)	95 (43.2)	
Yes	502 (57.5)	377 (57.7)	125 (56.8)	
Vaccination dose, n (%)				0.627
0	83 (9.5)	59 (9)	24 (10.9)	
1	31 (3.6)	21 (3.2)	10 (4.5)	
2	257 (29.4)	196 (30)	61 (27.7)	
3	502 (57.5)	377 (57.7)	125 (56.8)	
Symptom, n (%)				0.346
No	379 (43.4)	277 (42.4)	102 (46.4)	
Yes	494 (56.6)	376 (57.6)	118 (53.6)	
Fever, n (%)				0.203
No	721 (82.6)	546 (83.6)	175 (79.5)	
Yes	152 (17.4)	107 (16.4)	45 (20.5)	
Cough, n (%)				0.556
No	551 (63.1)	408 (62.5)	143 (65)	
Yes	322 (36.9)	245 (37.5)	77 (35)	
Sore throat, n (%)				0.409
No	716 (82.0)	531 (81.3)	185 (84.1)	
Yes	157 (18.0)	122 (18.7)	35 (15.9)	
Catarrhal symptoms, n (%)				0.076
No	827 (94.7)	613 (93.9)	214 (97.3)	
Yes	46 (5.3)	40 (6.1)	6 (2.7)	
Headache or myalgia, n (%)				0.15
No	790 (90.5)	585 (89.6)	205 (93.2)	
Yes	83 (9.5)	68 (10.4)	15 (6.8)	
Fatigue, n (%)				0.593
No	854 (97.8)	640 (98)	214 (97.3)	
Yes	19 (2.2)	13 (2)	6 (2.7)	
Co-morbidities, n (%)				0.623
No	714 (81.8)	537 (82.2)	181 (82.3)	
Yes	159 (18.2)	116 (17.8)	39 (17.7)	
Hypertension, n (%)				0.763
No	796 (91.2)	597 (91.4)	199 (90.5)	
Yes	77 (8.8)	56 (8.6)	21 (9.5)	
Diabetes, n (%)				0.65
No	843 (96.6)	629 (96.3)	214 (97.3)	
Yes	30 (3.4)	24 (3.7)	6 (2.7)	
Chronic bronchitis, n (%)				0.571
No	829 (95.0)	618 (94.6)	211 (95.9)	
Yes	44 (5.0)	35 (5.4)	9 (4.1)	
Smoking status, n (%)				0.089
No	632 (72.4)	483 (74)	149 (67.7)	
Yes	241 (27.6)	170 (26)	71 (32.3)	

PPQH group, Pupingqinghua group; LHQW group, Lianhuaqingwen group; BMI, body mass index. Continuous variables were compared with those of the non-parametric test. Categorical variables were compared by the χ^2^ test or Fisher’s exact tests.

## 3.2 Viral load shedding time and Ct value

**FIGURE 1:**
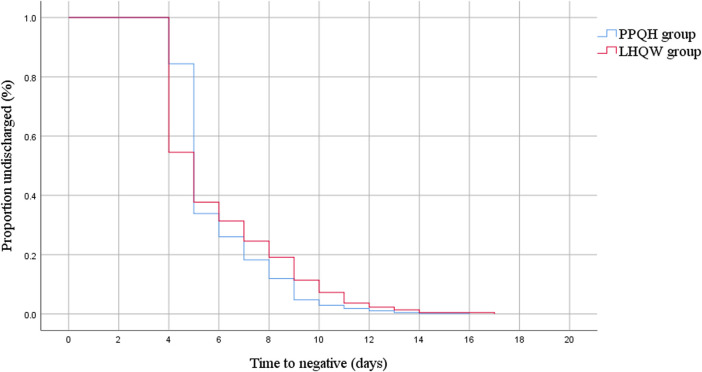
Kaplan–Meier estimate of the viral load shedding time between the two groups of SARS-CoV-2-infected patients.

The median viral load shedding time of 873 patients was 5.0 (5.0, 7.0) days, and for the PPQH group and LHQW group, it was 5.0 (5.0, 7.0) and 5.0 (4.0, 7.0), respectively. The viral load shedding time of the PPQH group was more concentrated and stable than that of the LHQW group (*p* < 0.01). After the treatment, the symptoms of the two groups were significantly improved. The symptom elimination rates in the PPQH group and LHQW group were 91.1 % and 88.6 %, respectively. There was no significant difference between the two groups (*p* > 0.05) (Table 2; Figure 1).

**TABLE 2:** Changes of the viral load shedding time between the two groups of SARS-CoV-2-infected patients.

	Total (*n* = 873)	PPQH group (*n* = 653)	LHQW group (*n* = 220)	*p* value
Viral load-shedding time (days), Median (IQR)	5.0 (5.0, 7.0)	5.0 (5.0, 7.0)	5.0 (4.0, 7.0)	0.001
Current symptoms, n (%)				0.341
Disappeared	790 (90.5)	595 (91.1)	195 (88.6)	
Not disappeared	83 (9.5)	58 (8.9)	25 (11.4)	

Continuous variables were compared by the non-parametric test. Categorical variables were compared by the χ^2^ test or Fisher’s exact tests.

**FIGURE 2:**
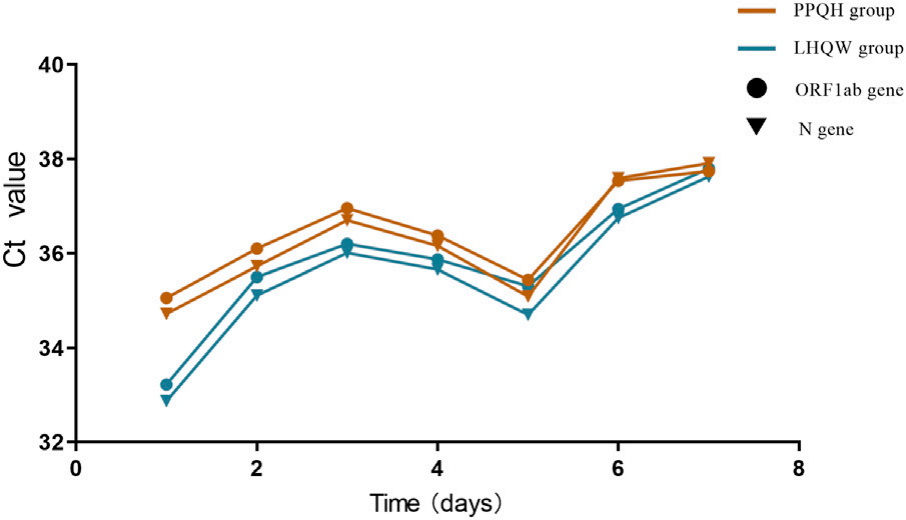
Comparison of Ct values of ORF 1 ab and N gene between the two groups of SARS-CoV-2-infected patients.

We counted the Ct value of the COVID-19 test results of 873 patients seven times, and the first test is carried out on the third day of hospitalization. After treatment with two drugs, the level of the ORF 1ab and N gene Ct value in the overall case showed an increasing trend, and the PPQH group was higher than the LHQW group at the first time (*p* < 0.01). At the fifth time, the Ct values of the two groups decreased slightly and then gradually increased; (Table 3; Figure 2);

**TABLE 3:** Comparison of the Ct values of the ORF1ab and N gene between the two groups of SARS-CoV-2-infected patients (x ± s). **p < 0.01. (X is the average value, i.e. X bar, and a horizontal line should be added at the top of X.

	ORF1ab gene		*p* value	N gene		*p* value
	PPQH group	LHQW group		PPQH group	LHQW group	
1	35.06 ± 6.84	33.22 ± 7.28	0.001**	34.72 ± 7.38	32.87 ± 7.67	0.001**
2	36.1 ± 5.98	35.5 ± 6.74	0.261	35.73 ± 6.49	35.11 ± 7.03	0.157
3	36.96 ± 5.11	36.2 ± 6.76	0.233	36.7 ± 5.63	36.01 ± 6.33	0.235
4	36.38 ± 5.16	35.87 ± 6.64	0.272	36.16 ± 5.83	35.66 ± 5.77	0.516
5	35.44 ± 5.21	35.31 ± 7.01	0.867	35.09 ± 6.03	34.7 ± 5.94	0.783
6	37.54 ± 3.67	36.94 ± 6.48	0.781	37.59 ± 4.16	36.75 ± 5.18	0.673
7	37.74 ± 3.04	37.8 ± 7.01	0.497	37.91 ± 3.7	37.63 ± 3.52	0.632

**p < 0.01. Continuous variables were compared by the non-parametric test.

### 3.3 Recovery time for symptoms of fever, sore throat, and cough

Linear regression analysis of 873 patients showed that age (*p* < 0.05), basic diseases such as hypertension (*p* < 0.01), and symptoms were independent risk factors for prolonging negative conversion days, fever (*p* < 0.05), cough (*p* < 0.01), sore throat (*p* < 0.01), catarrhal symptoms (*p* < 0.05), headache, or myalgia (*p* < 0.05) (Table 4).

**TABLE 4:** Correlation analysis of independent factors and COVID-19 test negative time.

	Ref	*p* value
Gender	0.18 (−0.11, 0.47)	0.227
Age	0.01 (0, 0.02)	0.015*
BMI	0.01 (−0.03, 0.05)	0.617
Vaccinated or not	−0.31 (−0.78, 0.17)	0.203
Three-dose vaccine or not	−0.11 (−0.39, 0.17)	0.438
Vaccination dose		
1	−0.41 (−1.28, 0.45)	0.345
2	−0.26 (−0.77, 0.26)	0.328
3	−0.32 (−0.81, 0.16)	0.19
Symptom	1.0091 (0.7379, 1.2804)	0.001**
Fever	0.82 (0.46, 1.18)	0.001**
Cough	0.76 (0.47, 1.04)	0.001**
Sore throat	1.06 (0.71, 1.42)	0.001**
Catarrhal symptoms	0.72 (0.11, 1.34)	0.022*
Headache or myalgia	0.53 (0.06, 1)	0.027*
Fatigue	0.39 (−0.56, 1.34)	0.418
Co-morbidities	0.25 (−0.11, 0.61)	0.177
Hypertension	0.67 (0.19, 1.16)	0.007**
Diabetes	−0.12 (−0.88, 0.64)	0.763
Chronic bronchitis	0.05 (−0.58, 0.69)	0.865
Smoking status	0.14 (−0.17, 0.45)	0.384

* *p* < 0.05.

** *p* < 0.01. The hazard ratios, two-sided 95% confidence intervals, and *p* value were estimated with the use of Cox regression with the baseline stratification factors as covariates.

**FIGURE 3:**
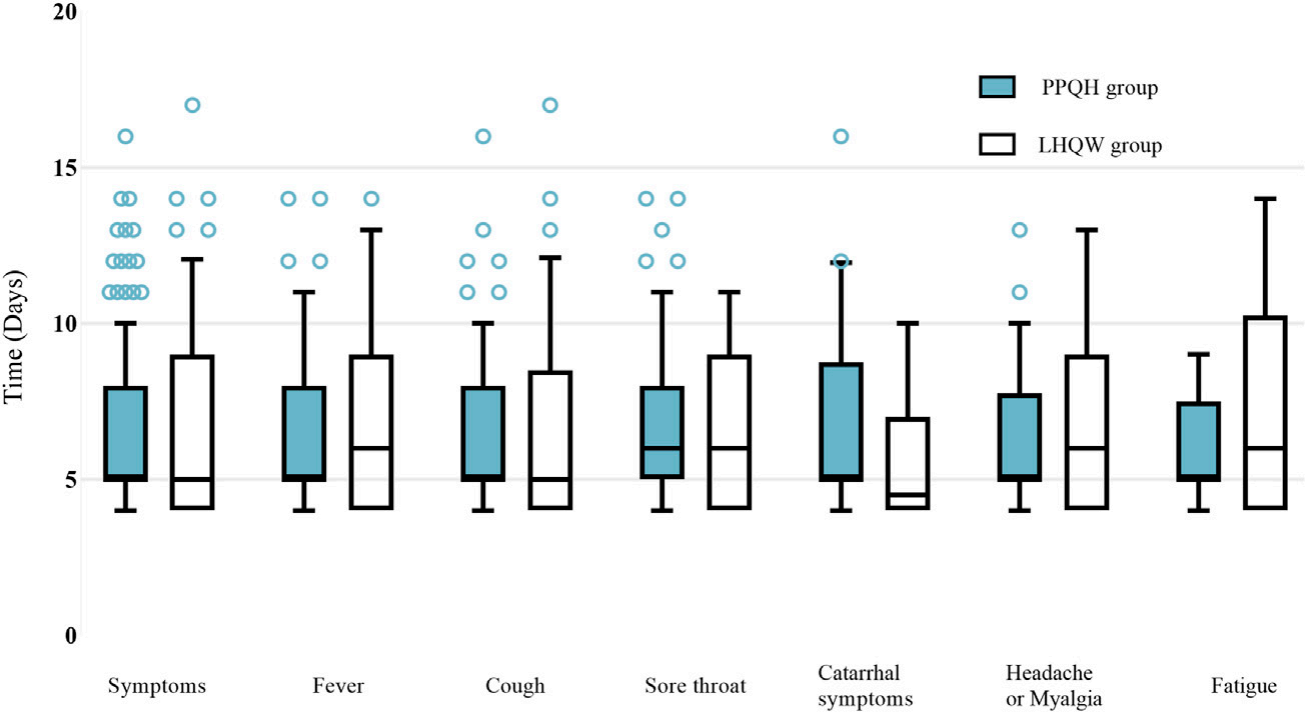
Comparison of the recovery time of symptoms between the two groups of SARS-CoV-2-infected patients.

In terms of symptom improvement, the recovery time in the PPQH group and LHQW group was similar, and that in the PPQH group was slightly shorter in the symptom of fever, headache or myalgia, and fatigue, but there was no statistical difference between the two groups. Combined with the regression analysis results, both the PPQH and LHQW groups can shorten the viral load shedding time and improve the symptoms (Table 5; Figure 3).

**TABLE 5:** Comparison of the recovery time of symptoms between the two groups of SARS-CoV-2-infected patients.

	Fever	Cough	Sore throat	Catarrhal symptoms	Headache or myalgia	Fatigue
PPQH group (days) median (IQR)	5.0 (5.0, 8.0)	5.0 (5.0, 8.0)	6.0 (5.0, 8.0)	5.0 (5.0, 8.2)	5.0 (5.0, 7.5)	5.0 (5.0.7.0)
LHQW group (days) median (IQR)	6.0 (4.0, 9.0)	5.0 (4.0, 8.0)	6.0 (4.0, 9.0)	4.5 (4.0, 5.8)	6.0 (4.0, 9.0)	6.0 (4.0, 9.0)
Z	0.174	0.268	0.79	2.358	−0.104	−0.275
*p* value	0.677	0.605	0.374	0.125	0.917	0.783

Continuous variables were compared by the non-parametric test.

### 3.4 COVID-19 test re-positive rate

The results of the COVID-19 retest of 873 patients after discharge and 7 days of isolation were statistically analyzed. Fifteen (3.2 %) patients showed re-positive results, including 10 (1.5 %) patients in the PPQH group and 5 (2.3 %) in the LHQW group. There was no statistical difference between the two groups (table 6).

**TABLE 6:** Comparison of the re-positive rate between the two groups of SARS-CoV-2-infected patients.

	Total (n = 873)	PPQH group (*n* = 653)	LHQW group (*n* = 220)	*p* value
Retest PCR result after 7 days, n (%)				0.548
Negative	858 (98.3)	643 (98.5)	215 (97.7)	
Positive	15 (1.7)	10 (1.5)	5 (2.3)	

Categorical variables were compared by the χ^2^ test or Fisher’s exact tests.

### 3.5 Inflammatory factors and lymphocyte subsets

Among the SARS-CoV-2 infected patients admitted to the cabin hospital, only a part of the patients were tested for hematological examination. Due to the small number of patients undergoing hematological examination in the LHQW group, we only included 27 patients who took the hematological examination and were treated with PPQH. The examinations were performed immediately after admission and before discharge, as shown in Table 7. The levels of IL-5, IL-6, and IL-10 of the patients after treatment with PPQH showed a downward trend compared with those before admission (*p* < 0.05), among which IL-5 decreased significantly (*p* < 0.01). The levels of NK cells and INF-α were decreased compared with those before treatment (*p* < 0.05), and the INF-α level was significantly decreased (*p* < 0.01). It may be suggested that PPQH can avoid organ damage caused by inflammation storms in the body and further turn into a severe disease as well as LHQW (Runfeng et al., 2020) (Tables 7, 8).

**TABLE 7:** Changes of inflammatory factors of SARS-CoV-2-infected patients.

	Before therapy	After therapy	Before–after	t/Z	*p* value
IL-1β (pg/ml)	0.89 ± 0.35	1.06 ± 0.65	−0.16 ± 0.63	−1.311	0.202
IL-2 (pg/ml)	0.88 (0.65.1.12)	0.92 (0.58.1.31)	−0.37 (−0.64.0.42)	−1.130	0.258
IL-4 (pg/ml)	1.25 (1.07.1.48)	1.24 (0.80.1.86)	0.01 (−0.73.0.36)	−0.610	0.542
IL-5 (pg/ml)	0.53 ± 0.17	0.4 ± 0.16	0.14 ± 0.23	3.044	0.005**
IL-6 (pg/ml)	1.98 (1.28.3.27)	1.39 (1.1.1.96)	0.42 (−0.30.2.14)	−2.286	0.022*
IL-8 (pg/ml)	1.76 (1.30.2.42)	1.64 (1.15.2.33)	−0.08 (−0.54.0.90)	−0.140	0.889
IL-10 (pg/ml)	2.78 ± 1.5	1.86 ± 0.97	0.91 ± 1.85	2.505	0.019*
IL-12p70 (pg/ml)	0.47 ± 0.48	0.62 ± 0.51	−0.15 ± 0.6	−1.290	0.209
IL-17A (pg/ml)	1.92 ± 1.16	1.85 ± 1.74	0.07 ± 2.56	0.143	0.887

* *p* < 0.05.

** *p* < 0.01. IL-1β, interleukin 1β; IL-2, interleukin 2; IL-4, interleukin 4; IL-5, interleukin 5; IL-6, interleukin 6; IL-8, interleukin; IL-10, interleukin 10; IL-12p70, interleukin 12p70; IL-17A, interleukin 17A. Continuous variables were compared by the non-parametric test.

**TABLE 8:** Changes of lymphocyte subsets of SARS-CoV-2-infected patients (x ± s ). (X is the average value, i.e., X bar, and a horizontal line should be added at the top of X.

	Before therapy	After therapy	Before–after	t/Z	*p*
NK cell (%)	22.89 ± 8.47	19.62 ± 7.51	3.27 ± 5.71	2.746	0.012*
Th cell (%)	37.78 ± 8.15	40.48 ± 8.30	−2.70 ± 5.44	−2.384	0.026*
Ts cell (%)	22.03 (19.17.27.2)	24.94 (18.61.27.24)	−0.89 (−2.30.0.96)	−1.688	0.091
B cell (%)	11.16 ± 5.09	11.84 ± 4.62	−0.68 ± 2.82	−1.128	0.271
TNF-α (pg/ml)	1.63 ± 0.6	1.95 ± 1.08	−0.32 ± 1.06	−1.403	0.174
INF-α (pg/ml)	1.6 ± 1.2	0.67 ± 0.42	0.93 ± 1.31	3.307	0.003**
INF-γ (pg/ml)	1.2 ± 0.29	1.2 ± 0.38	0.01 ± 0.52	0.063	0.95

* *p* < 0.05.

** *p* < 0.01. NK, cell, natural killer cell; Th cell, helper T cell; T cell, suppressor T cell; B cell, B lymphocyte; TNF-α, Tumor Necrosis Factor-α; INF-α, Interferon-α; INF-γ, Interferon-γ. Continuous variables were compared by the non-parametric test.

### 3.6 Liver and kidney function

After treatment of PPQH, the levels of AST, ALT, eGFR, and creatinine in 27 patients showed no significant differences compared with those before treatment (*p* > 0.05), while the level of γ-GTT decreased significantly (*p* < 0.01), suggesting that PPQH did not have hepatorenal toxicity (Table 9).

**TABLE 9:** Liver and kidney function indexes in the PPQH group of SARS-CoV-2-infected patients.

	AST (U/L)	ALT (IU/L)	γ-GTT (U/L)	eGFR (ml/min)	Creatinine (umol/L)
Before therapy	27.85 ± 14.26	28.41 ± 26.18	22(14. 48)	109 (99, 115)	54(49.5. 71.5)
After therapy	25.54 ± 10.47	26.48 ± 21.89	19(13.5.39)	108 (98.5, 119)	61(54. 73.5)
Before—after	2.31 ± 8.38	1.92 ± 14.27	1 (−1.3)	2 (0. 3.5)	−2 (−5. 0.5)
t/Z	1.405	0.688	−2.847	−0.655	−1.677
*p* value	0.172	0.498	0.004**	0.512	0.094

**p < 0.01. AST, aspartate aminotransferase; ALT, alanine aminotransferase; γ-GTT, gamma-glutamyl transpeptidase; eGFR, Glomerular Filtration Rate (EGFR_EPI_Cr). Continuous variables were compared by the non-parametric test.

### 3.7 Adverse reactions

After the treatment, adverse events of varying degrees occurred in both the PPQH group and LHQW group, of which the main adverse reactions were gastrointestinal reactions; 52 (6.0 %) patients occurred in the total included population, of which 31 (4.7 %) patients occurred in the PPQH group, which was lower than that of the LHQW group in 21 (9.5 %) patients (*p* < 0.05); and the result may be related to the application of heat-clearing drugs in the LHQW group. There were four (0.5 %) patients with a red itching skin allergic reaction, three (0.5 %) patients in the PPQH group, and one (0.5 %) patient in the LHQW group. After drug withdrawal, the allergic reactions were improved, no severe allergic reaction occurred, and there was no statistically significant difference between the two groups (*p* > 0.05). Some patients had other adverse events such as palpitations, dizziness, and profuse sweating, but none seriously affected their vital signs; among them, eight (1.2 %) patients were in the PPQH group and 13 (5.9 %) patients were in the LHQW group. The number of patients in the PPQH group with adverse reactions was less than that in the LHQW group, with a statistical difference (*p* < 0.01) (Table 10).

**TABLE 10:** Comparison of the adverse reaction rates between the two groups of SARS-CoV-2-infected patients.

Variables	Total (n = 873)	PPQH group (n = 653)	LHQW group (*n* = 220)	*p* value
Gastrointestinal reaction, n (%)				0.015*
No	821 (94.0)	622 (95.3)	199 (90.5)	
Yes	52 (6.0)	31 (4.7)	21 (9.5)	
Skin allergies, n (%)				1
No	869 (99.5)	650 (99.5)	219 (99.5)	
Yes	4 (0.5)	3 (0.5)	1 (0.5)	
Other adverse reactions, n (%)				0.001**
No	852 (97.6)	645 (98.8)	207 (94.1)	
Yes	21 (2.4%)	8 (1.2)	13 (5.9)	

* *p* < 0.05.

** *p* < 0.01. Categorical variables were compared by the χ^2^ test or Fisher’s exact tests.

## 4 Discussion

COVID-19 is an acute respiratory infectious disease caused by SARS-COV-2, which a novel plus strand RNA Coronavirus. Its main clinical manifestations include fever, cough, myalgia, fatigue, and dyspnea. Some patients may also experience symptoms of headache, dizziness, diarrhea, nausea, and vomiting (Li et al., 2020a; Wang et al., 2022); severe acute respiratory distress syndrome (ARDS) and multiple organ failure (MOF) may result in death (Li et al., 2020b). The disease has the characteristics of strong infectivity, rapid spread, and widespread susceptibility among the population, causing severe harm to all countries in the world.

In the prevention and treatment of COVID-19, the National Health Commission of the People’s Republic of China has established the treatment policy of combining traditional Chinese and Western medicines, and TCM has shown significant advantages in treating COVID-19. As the recommended drug for treatment of COVID-19 in China, LHQW is a traditional Chinese medicine prescription composed of 13 kinds of traditional Chinese herbs, including *Lonicera japonica Thunb. [*Caprifoliaceae*]* (Jinyinhua), *Forsythia suspensa (Thunb.) Vahl [*Oleaceae*]* (Lianqiao), *Prunus armeniaca L. [*Rosaceae*]* (Xingren), *Ageratum conyzoides L. [*Asteraceae*]* (Huoxiang), *Strobilanthes cusia (Nees) Kuntze [*Acanthaceae*]* (Banlangen), *Ephedra sinica Stapf [*Ephedraceae*]* (Mahuang), *Houttuynia cordata Thunb. [*Saururaceae*]* (Yuxingcao), and *Rhodiola rosea L. [*Crassulaceae*]* (Hongjingtian). It is widely used in treatment of influenza combined with famous prescriptions of three dynasties from the Eastern Han Dynasty to the Ming and Qing Dynasties (Liang et al., 2021). Before the COVID-19 pandemic, LHQW had been approved to treat public health emergencies such as SARS, H1N1, and H3N2 influenza viruses, and clinical trials showed significant efficacy (Tao et al., 2013; Gao et al., 2020). During the outbreak of COVID-19, studies on the efficacy and safety of LHQW in the treatment of COVID-19 patients showed that the recovery rate and chest CT improvement rate in the LHQW treatment group were significantly higher than those in the control group. The recovery time was significantly shortened, and the mechanism may be related to inhibition of IL-6, IP-10, and TNF-α and improvement of inflammatory cell infiltration (Li et al., 2020c; Runfeng et al., 2020; Wan, 2020; Hu et al., 2021; Shen and Yin, 2021). In this study, LHQW can improve the clinical symptoms of patients with SARS-COV-2 infection, which is consistent with previous studies.

Inflammatory storm is a systemic inflammatory response triggered by various factors, including infection and drugs, and is characterized by a sharp increase in the levels of a large number of pro-inflammatory cytokines. Evidence from a wide range of sources suggests that SARS‐CoV‐2 infection induces an excessive inflammatory response and cytokine storm (CS), and CS is a vital factor in the conversion of patients with COVID-19 from mild to severe and critical illness (Gu et al., 2022). After infection with SARS-COV-2, the innate immune system is triggered, and various inflammatory pathways are activated, among which NLRP/IL-1β, JAK/STAT, TNF-α/NF-κB, and other pathways have been confirmed to be closely related to SARS-CoV-2 infection (Jiang et al., 2020). The levels of various pro-inflammatory cytokines (interleukin [IL]‐6, IL‐1β, IL‐2R, IL‐8, tumor necrosis factor‐α [TNF‐α], and interferon [IFN]‐γ) and immune cells (monocytes, macrophages, neutrophils, dendritic cells, NK cells, CD4^+^, and CD8^+^ T cells) are elevated, resulting in excessive activation of the immune system and occurrence of inflammatory CS. Subsequently, the host immune system initiates protective immune regulation, releasing regulatory cytokines, such as IL-10 and TGF-β, to combat immune hyperstimulation. IL-6 and TNF-α may be the most critical factors causing CS; IL-6 and IL-10 levels can be used as a primary index for predicting COVID-19 outcomes (Leong et al., 2006). In this study, after treatment with PPQH, pro-inflammatory cytokines (IL-5, IL-6, and INF-α), regulatory cytokines (IL-10), and innate immune cells (NK cells) showed a downward trend compared with that before treatment (*p* < 0.05).

In the formulation of the PPQH prescription, Yinchenhao decoction (YCHD) is included. YCHD is a classic prescription in TCM that is mainly used for internal stasis heat and jaundice, and it is also used for internal medicine, surgery, and pediatrics (Li et al., 2017). YCHD was found to reduce AST (aspartate aminotransferase), interferon-γ, and Fas gene levels and increase the level of interleukin IL-4 and IL-6 levels in the serum, reducing the inflammatory response (Cai et al., 2019). In addition, organic acid component, as the main active ingredient of pugongying, can improve acute tracheobronchitis by downregulating the protein expression levels of TNF-α and IL-6 (Yang et al., 2016). The main chemical constituents of Fuping, the flavones apigenin and luteolin, can suppress inflammatory responses by mediating neutrophil apoptosis (Lucas et al., 2013); In addition, Cangshu, Zhizi, and Huangqi have also been confirmed to have anti-inflammatory and anti-oxidant effects (Xiao et al., 2017; Kulma et al., 2021; Li et al., 2022). Combined with the aforementioned results, it may be suggested that PPQH plays an anti-SASR-COV-2 effect through its antiviral and anti-inflammatory cytokine storm functions; however, further research is still needed.

Based on the theory of TCM, the pathogenesis of SARS-COV-2 infection (Omicron) is mainly due to dampness. Prolonged dampness will heat up and cause fever symptoms. On the other hand, dampness blocks Qi and causes lung qi to increase, leading to cough. This pathogenesis explains that fever and cough are the main clinical symptoms of patients with SARS-CoV-2 infection (Guan et al., 2020). PPQH takes into account both heat-clearing and dampness removal and LHQW in relieving symptoms and the viral load shedding time.

In this study, the median time of the viral load shedding time of both the PPQH group and LHQW group was 5 days, which was less than the average time reported by other studies (Shen et al., 2022), and there was no difference in symptom recovery and re-positive rate between the two groups. In the terms of safety indicators, 4.7 % of patients had gastrointestinal reactions and 0.5 % of patients had skin allergies in the PPQH group. The incidence rate of allergy, palpitation, dizziness, sweating, and other adverse events of the PPQH group was 1.2 %, which suggested that PPQH was safe and mild.

This study has the following limitations: one of the limitations is the sample size of the hematological indicators. Due to the small sample size, we only compared the hematological indexes of the PPQH group; it can only partially explain the anti-inflammatory effect and safety of the liver and kidney functions of PPQH. In addition, this study is a single-center retrospective study; we also need to conduct a multi-center randomized controlled trial to provide higher-level clinical evidence. In conclusion, PPQH can shorten the length of hospital stay and improve the clinical symptoms of patients with SARS-COV-2 (Omicron) and has a good safety profile.

## Data Availability

The raw data supporting the conclusions of this article will be made available by the authors, without undue reservation.

## Abbreviations

ARDS: acute respiratory distress syndrome; COVID-19: Coronavirus disease 2019; LLQH: lianhuaqingwen MOF: multiple organ failure PPQH: Pupingqinghua prescription TCM: traditional Chinese Medicine VOC: variant of concern CS: cytokine storm YCHD: Yinchenhao decoction AST: aspartate aminotransferase
